# A comparison of the ability of the human IgG1 allotypes G1m3 and G1m1,17 to stimulate T-cell responses from allotype matched and mismatched donors

**DOI:** 10.1080/19420862.2015.1128605

**Published:** 2016-01-29

**Authors:** Carl I. Webster, Christine J. Bryson, Edward A. Cloake, Tim D. Jones, Mark J. Austin, Anette C. Karle, Sebastian Spindeldreher, David C. Lowe, Matthew P. Baker

**Affiliations:** a MedImmune Ltd, Milstein Building, Granta Park, Cambridge, CB21 6GH, United Kingdom; b Antitope Ltd (An Abzena company), Babraham Research Campus, Babraham, Cambridge, CB22 3AT, United Kingdom; c Novartis Pharma AG, Klybeckstrasse 141, CH-4057 Basel, Switzerland

**Keywords:** Allotype, Epitope, HLA, IgG, Immunogenicity, MAPPs, MHC, T-cell

## Abstract

The immunogenicity of clinically administered antibodies has clinical implications for the patients receiving them, ranging from mild consequences, such as increased clearance of the drug from the circulation, to life-threatening effects. The emergence of methods to engineer variable regions resulting in the generation of humanised and fully human antibodies as therapeutics has reduced the potential for adverse immunogenicity. However, due to differences in sequence referred to as allotypic variation, antibody constant regions are not homogeneous within the human population, even within sub-classes of the same immunoglobulin isotype. For therapeutically administered antibodies, the potential exists for an immune response from the patient to the antibody if the allotype of patient and antibody do not match. Allotypic distribution in the human population varies within and across ethnic groups making the choice of allotype for a therapeutic antibody difficult. This study investigated the potential of human IgG1 allotypes to stimulate responses in human CD4^+^ T cells from donors matched for homologous and heterologous IgG1 allotypes. Allotypic variants of the therapeutic monoclonal antibody trastuzumab were administered to genetically defined allotypic matched and mismatched donor T cells. No significant responses were observed in the mismatched T cells. To investigate the lack of T-cell responses in relation to mismatched allotypes, HLA-DR agretopes were identified via MHC associated peptide proteomics (MAPPs). As expected, many HLA-DR restricted peptides were presented. However, there were no peptides presented from the sequence regions containing the allotypic variations. Taken together, the results from the T-cell assay and MAPPs assay indicate that the allotypic differences in human IgG1 do not represent a significant risk for induction of immunogenicity.

## Introduction

Early attempts to develop monoclonal antibodies (mAbs) therapeutically were based on the then newly emerged hybridoma technology,^1^ which enabled the production of large quantities of mouse mAbs. Administration of these mAbs most often resulted in severe immune responses from the patient to the antibody. As antibody technology advanced, mouse monoclonal therapeutics were mostly replaced with chimeric antibodies in which the mouse constant domains are replaced with human sequences, humanized antibodies that contain human framework residues with mouse complementarity-determining regions, and ultimately fully human antibodies from display technologies or mice transgenic for human antibody sequences.^2-6^ Over the past 30 years, mAbs have become established as a major class of therapeutic molecule, with over 400 antibodies entering clinical development during this time.^7^ As the content of non-human sequences in therapeutic antibodies has decreased, so too has the incidence of immunogenicity.^8^ However, immune reactions to clinically administered antibodies have not disappeared completely, even in the case of fully human antibodies. The source of this immunogenicity is frequently, but not exclusively, the variable domains of the antibodies.^9^ In these instances immunogenicity can be explained by anti-idiotypic responses, responses due to differences in germline expression, or break of tolerance. Immune reactions to the Fc domain of human mAbs used therapeutically are less common, but could theoretically still occur.^10^ While the antigen combining domains of antibodies are highly variable, the remainder of the antibody has relatively invariable sequence. However, among the human population the constant domains of a given antibody isotype retain a small degree of variability. This phenotypic heterogeneity was identified many years ago as a consequence of the anti-immunoglobulin responses seen as a component of rheumatoid factors,^11^ as a result of immune reactions to immunoglobulins contained in blood transfusions ^12^ and maternal-fetal incompatibility.^13,14^ These differences in serological properties were termed allotypes ^15^ and have been attributed to amino acid differences in the constant domains of the immunoglobulins. The number and position of these substitutions varies according to the antibody class, isotype and protein chain. For example, there is a single allotype of IgG2, but 13 for IgG3.^16^ The nomenclature for allotypes is based on alphabetical or numerical designations agreed by the World Health Organization.^17^ That these allotypes were detected serologically is a clear indication of an immune response to antibodies from one allotype administered to an individual carrying a different allotype. This therefore has the potential to affect the use of antibody drugs of one allotype in patients genetically lacking that allotype.

One of the most commonly used sub-classes of the IgG isotype for therapeutic antibodies is an IgG1,^7,18^ which has 4 well-characterized allotypes *a, x, f* and *z* (G1m1, G1m2, G1m3, and G1m17, respectively).^16^ The molecular basis of G1m1 was determined in the 1960s ^19,20^ and that of G1m3 and G1m2 several years later.^21^ The G1m3 and G1m17 allotypes are mutually exclusive as they both arise through an amino acid substitution at position CH1 120 (arginine and lysine, respectively) and the G1m1 allotype differs from the null allotype (nG1m1) at positions CH3 12 and 14 (IMGT numbering; www.imgt.org) where glutamate and methionine are replaced by aspartate and leucine. The frequency, and in some instances the presence, of these allotypes within the population varies according to ethnic group.^22^

Biopharmaceutical companies are therefore faced with a difficult choice in determining the allotype on which to base their therapeutic antibodies. The result is an inconsistent mix of different and sometimes hybrid IgG1 allotypes in the clinic.^10^ Recent studies have attempted to investigate whether anti-drug antibody (ADA) responses seen against therapeutic mAbs could be attributed to a mismatch between the allotype of the antibody and the patients receiving it. The results indicated that there was no difference in the antibody responses to the Fc domain among patients with an allotype matched to infliximab and those with a mismatch,^23^ and similar results were found with patients receiving adalimumab, although it was observed that rheumatoid arthritis patients with the G1m17 allotype were more likely to develop anti-adalimumab antibodies.^24^

An adaptive immune response against exogenous antigens (such as protein therapeutics) leading to the production of isotype switched, high-affinity ADAs requires ‘help’ in the form of co-stimulation from CD4^+^ T helper cells. Linear peptide T-cell epitopes that bind to the HLA class II binding groove are generated from the antigen after uptake and processing by antigen presenting cells (APCs). T-cell epitopes presented in the context of HLA class II are recognized via binding of the cognate T-cell receptor (TCR) on the T cell, which, in combination with costimulatory signals, results in T-cell activation. Activated T cells are able to drive downstream events including maturation of B cells into memory and antibody secreting plasma B cells. Therefore, it is possible that an allotype difference could generate a novel T-cell epitope in ‘allotype mismatched’ patients and drive the T-cell response into stimulating an anti-therapeutic humoral response (that may be directed against multiple B-cell epitopes distal to the T-cell epitope).

Assessment of the potential for immunogenicity via T-cell epitope analysis is becoming increasingly important during the pre-clinical development of protein therapeutics. Human *ex vivo* T-cell assays have been developed using community blood donors carefully selected based on HLA class II haplotypes to enable the quantification of T-cell responses against protein therapeutics. Such T-cell assays have been shown to correlate with the incidence of clinical immunogenicity and therefore provide a useful tool in developing safer, efficacious protein therapeutics.^25^

Using *ex vivo* T-cell assays and MHC associated Peptide Proteomics (MAPPs) assays,^26-28^ we have investigated T-cell responses to G1m1,17 and G1m3 allotypes of trastuzumab using T cells from donors homozygous for each allotype and identified HLA-DR presented peptides using monocyte-derived dendritic cells (DCs). We conclude that there is no difference in T-cell responses between allotype matched and mismatched donors to the 2 allotypes of trastuzumab and no peptides containing the allotype sequences are presented in the context of HLA-DR, indicating that differences in antibody allotype do not lead to increased immunogenicity.

## Results

### Identification of G1m allotypes and donor selection

PCR and sequence analysis were used to test genomic DNA prepared from 570 community blood donors for G1m allotype. Through visual analysis of chromatographs, the heterozygous allotype manifested itself as overlapping peaks corresponding to 2 different nucleotides each encoded by alternate G1m alleles at the CH1 and CH3 loci (Fig. S1A). In contrast, the homozygous allotype manifested itself as a single chromatograph peak, corresponding to 2 identical, overlapping G1m alleles (Fig. S1B). 51 donors were found to be homozygous G1m1/G1m17 and 259 homozygous nG1m1/G1m3, giving a frequency in the sampled population of 8.9% and 45.4%, respectively. Occasional recombinant events were identified within the panel of tested donor samples. This included 4 donors that were homozygous G1m17 at their CH1 locus and heterozygous G1m1/nG1m1 at their CH3 locus, plus 3 donor samples that were heterozygous G1m17/G1m3 at their CH1 locus and homozygous nG1m1 at their CH3 locus.

**Figure 1.**
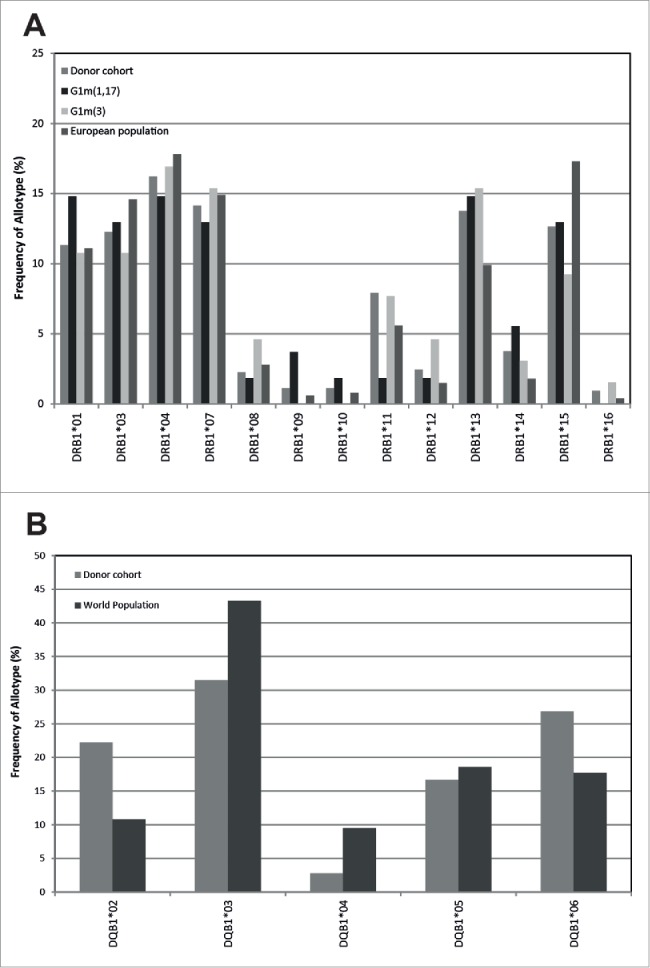
Comparison of the frequency of donor DRB (a) and DQB (b) allotypes expressed in the European population against the whole study donor cohort (n = 68), and donors expressing the homozygous G1m3 allotype (n = 37) and donors expressing the homozygous G1m1,17 allotype (n = 31)

All 570 donors were low resolution tissue typed for HLA- DR class II allotypes, and this data was used to select a population of 37 homozygous G1m3 donors and 31 homozygous G1m1,17 donors for the time course T-cell assays whose HLA-DR distribution frequency was representative of that observed in the European population (Fig. 1A) (http://www.ncbi.nlm.nih.gov/gv/mhc/main.fcgi?cmd=init). All major HLA-DR allotypes (individual allotypes with a frequency >5% expressed in the European population) were well represented. All 68 donors selected for the time course T-cell assays were also low resolution tissue typed for HLA-DQ; Fig. 1B shows the frequency distribution of HLA-DQ allotypes in the study cohorts in comparison to that found in the European population (http://www.ncbi.nlm.nih.gov/gv/mhc/main.fcgi?cmd=init). Again, the donor sets were found to be evenly matched.

### Healthy donor T cell responses to IgG1 G1m3-trastuzumab and G1m1,17-trastuzumab

**Figure 2.**
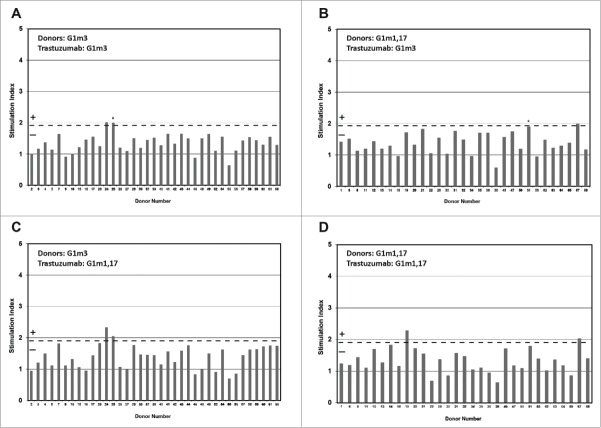
Healthy donor T-cell proliferation to IgG1 G1m3-trastuzumab and G1m1,17-trastuzumab. T-cell proliferation was measured from bulk cultures of PBMC on days 5, 6, 7 and 8. Figure shows the maximum stimulation index over the 4 day period. The threshold for positive T-cell proliferation (SI ≥ 1.90, p < 0.05) is indicated by red dotted line. Borderline responses (SI = 1.90-1.95, p < 0.05) are indicated (*). Donors expressing homozygous G1m3 (a and c) and G1m1,17 (b and d) were tested against G1m3-trastuzumab (a and b) and G1m1,17-trastuzumab (c and d)

**Figure 3.**
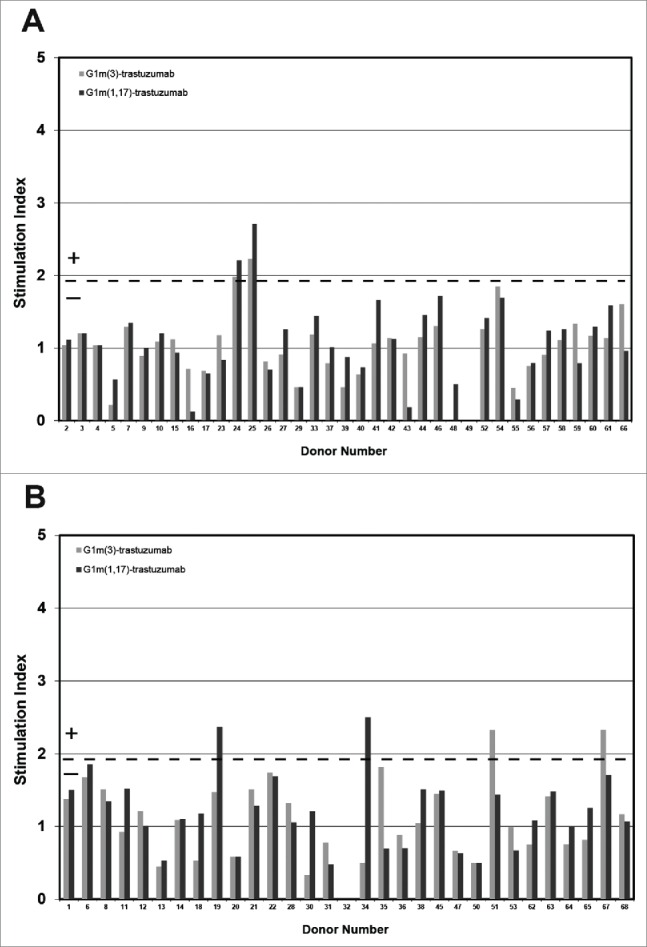
Healthy donor IL-2 ELISpot responses to IgG1 G1m3-trastuzumab and G1m1,17-trastuzumab. Replicate cultures were incubated in the presence of test samples for a total of 8 days (in parallel to the proliferation assays) prior to detection of secreted IL-2. The threshold for positive IL-2 ELISpot responses (SI ≥ 1.90, p < 0.05) is indicated by the dotted line. Borderline responses (SI = 1.90-1.95, *p* < 0.05) are indicated (*). (a) homozygous G1m3 donors and (b) homozygous G1m1,17 donors. No ELISpot data was obtained from donors 32 and 49.

In the time course assay, PBMC from 68 healthy donors were incubated with G1m3-trastuzumab and G1m1,17-trastuzumab in bulk 2 ml cultures over an 8-day period. The cultures were sampled and T-cell proliferation measured on days 5, 6, 7 and 8 (Fig. 2A-D, see also Table S1 and Table S2 for cpm data). Donors expressing the homozygous G1m3 allotype are shown in Fig. 2A and C, and those expressing the homozygous G1m1,17 allotype are shown in Fig. 2B and D, while the results for G1m3-trastuzumab are shown in Fig. 2A and B, and those for G1m1,17-trastuzumab in Fig. 2C and D. The IL-2 ELISpot assay was performed in parallel (Fig. 3, see also Table S3 for spw data) where G1m3 and G1m1,17 donors are shown in Fig. 3A and B, respectively. Analysis of the frequency of positive T-cell responses showed that all the antibody samples stimulated a positive response in a small proportion of donors in both proliferation and IL-2 ELISpot assays. The overall correlation between responses observed in proliferation and IL-2 ELISpot assays was high for all samples tested (87.5%), although in one donor (no. 34) IL-2 ELISpot responses were seen in the absence of proliferation and in another (no. 67) proliferation was seen in the absence of an ELISpot response. The ELISpot assay captures IL-2 throughout the duration of the assay (1–8 days) as opposed to specific time points (days 5, 6, 7 and 8); therefore differences can arise due to the kinetics of the IL-2 and proliferation responses or can be caused by the activation of cell subsets that undergo limited proliferation. Responding donors were therefore defined as those that mounted a positive response to each sample in *both* IL-2 ELISpot *and* proliferation assays. This high degree of correlation was also observed for keyhole limpet hemocyanin (KLH)-specific T-cell responses with 71% of donors producing matching responses against KLH in both proliferation and IL-2 ELISpot assays (typical range in the PBMC based T-cell assays is 70–80%, not shown). The strong response against KLH is in line with the high number of potential T-cell epitopes within the sequence of KLH observed in the MAPPs assay (Fig. S2). In the homozygous G1m3 allotype donors, 5% responded positively (SI ≥ 1.90 *p* < 0.05) in both the ELISpot and proliferation assays to G1m3-trastuzumab, and in the homozygous G1m1,17 allotype donors, 3% responded positively to G1m1,17-trastuzumab (Table 2). This frequency of response corresponds with previous data from *ex vivo* time course T-cell assays where frequency of T-cell responses against trastuzumab in different cohorts of donors ranged from 4–6%.^25,29^ Mismatching homozygous G1m3 allotype donors with G1m1,17-trastuzumab gave a combined frequency of positive response in the proliferation and ELISpot assay of 5%. Inversely, the homozygous G1m1,17 donors incubated with the G1m3-trastuzumab gave a combined responding frequency of 6% in the ELISpot and proliferation assays (Table 2). The results show that mismatching the donor allotype with the allotype of the trastuzumab antibody gave a similar positive response frequency to the matched donors.

**Table 1.** Summary of human IGγ1 (G1m) allotypes and their associated genotypes (From: http://www.imgt.org).

G1m allotypes		Amino acid positions^a^		
CH1	CH3	CH1	CH3	
		120	12	14
		(97)	(16)	(18)
		*214*	*356*	*358*
G1m17	G1m1	Lys	Asp	Leu
		aAa	gaT	Ctg
G1m3	nG1m1	Arg	Glu	Met
		aGa	gaG	Atg

a Amino acid numbers (bold) are defined according to IMGT unique numbering for C-DOMAIN^36^; http://www.imgt.org). Numbers in parentheses define exons. Numbers in italics define Eu numbering. ^37^ The G1m17 allotype (Lys at position 120; CH1) and G1m1 allotype (Asp and Leu at positions 12 and 14, respectively; CH3) are both found on IGHG1*01 and IGHG1*2 alleles. The G1m3 allotype (Arg at position 120; CH1) and nG1m1 (Glu and Met at positions 12 and 14, respectively) are found on IGHG1*03 allele. Upper case letter within 3 letter amino acid code indicates polymorphic nucleotide.

**Table 2.** Summary of G1m3-trastuzumab and G1m1,17-trastuzumab specific T-cell proliferation and IL-2 ELISpot responses in the homozygous G1m3 and G1m1,17 donor cohorts. Frequency (expressed as a percentage of the donor cohort) of positive proliferation responses (SI ≥ 1.9, significant *p* < 0.05) and IL-2 responses (SI ≥ 1.9, significant *p* < 0.05).

Donors	Homozygous G1m3		Homozygous G1m1,17	
Sample	G1m3 Trastuzumab	G1m1,17 Trastuzumab	G1m3 Trastuzumab	G1m1,17 Trastuzumab
% Proliferation	5%	5%	6%	6%
% ELISpot	5%	5%	6%	6%
% Proliferation and ELISpot	5%	5%	6%	3%

### Healthy donor T cell responses to peptides spanning the IgG1 allotypic variations

**Figure 4.**
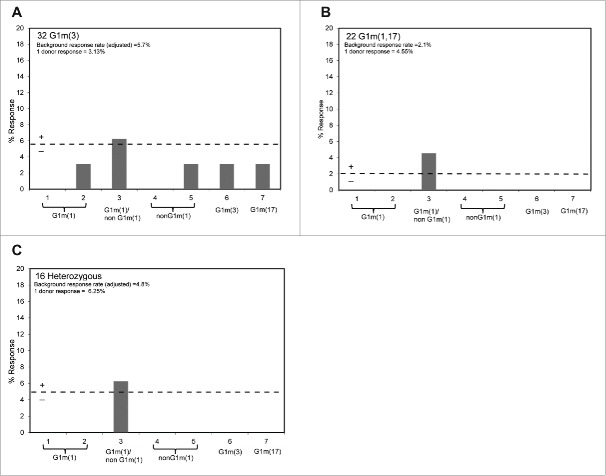
Healthy donor proliferation responses to peptides containing: G1m1 (peptides 1 and 2), nG1m1 (peptides 4 and 5), G1m3 (peptide 6) and G1m17 (peptide 7). Replicate cultures were incubated in the presence of peptides for a total of 7 days prior to assay for proliferation with groups of: (a) homozygous G1m3 donors, (b) homozygous G1m1,17 donors and (c) heterozygous donors. The background threshold for positive (SI ≥ 1.90, p < 0.05) proliferation is indicated by the dotted line (average responses +2 SD).

A total of 22 G1m1,17 homozygous, 32 G1m3 homozygous and 16 G1m1,17/G1m3 heterozygous individuals were used to test whether peptides spanning the allotypic variations comprising the different IgG1 allotypes could stimulate T-cell proliferation. The results in Fig. 4 show that for all 3 donor cohorts, none of the peptides that spanned the different IgG1 allotype sequences stimulated T-cell proliferation above background. However, a very weak T-cell proliferation response (one donor responded in each of the G1m1,17 homozygous and G1m1,17/G1m3 heterozygous cohorts, and 2 donors responded in the G1m3 homozygous cohort) was detected against peptide 3 (overlapping peptides 1, 2, 4 and 5) that comprised human germline sequence present in all homozygous and heterozygous individuals. This suggests the presence of a weak cryptic T-cell epitope in human germline IgG1 at this location that is not dependent upon allotypic variation. These data are consistent with the observations obtained by mismatching donors with different IgG1 allotypes of trastuzumab where no enhanced T-cell responses were detected.

### Peptides presented by DCs loaded with IgG1 G1m3-Trastuzumab and G1m1,17-Trastuzumab

Due to the limitation of available cells per donor, the MAPPs assay was performed on a different panel of donors than the T-cell assays. A total of 35 donors (12 G1m3 homozygotes, 7 G1m1,17 homozygotes and 16 heterozygotes) were analyzed using the MAPPs assay.^26-28,30^ Immature DCs were loaded with either G1m3 or G1m1,17 trastuzumab or KLH and media controls followed by stimulation to a mature phenotype. Naturally presented peptides were eluted from the cells and analyzed by liquid chromatography-mass spectrometry. HLA-DR–associated peptides can originate from various regions of a protein and typically occur as multiple length variants that share the same HLA-DR binding core and form a “cluster." Although theoretically every cluster has the potential to be recognized as a T-cell epitope, not every cluster may effectively induce a T-cell response.

**Figure 5.**
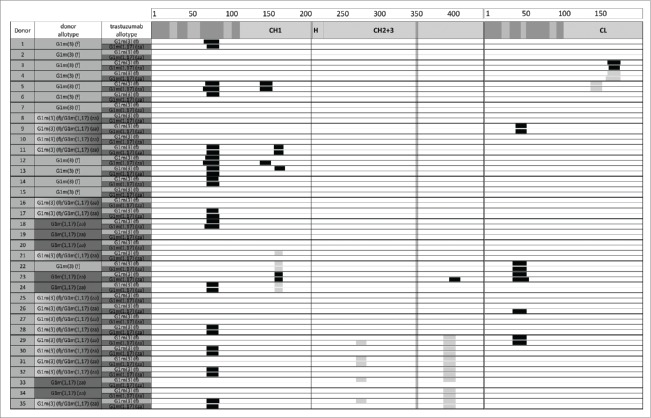
HLA-DR–associated peptides produced by 5 × 10^6^ dendritic cells from 35 different donors exposed separately to G1m1,17 and G1m3 allotypes of trastuzumab. HLA-DR–associated peptides can originate from various regions of a protein typically occurring as multiple length variants that share the same HLA-DR binding core and form a “cluster." Clusters are indicated as black boxes, CDRs are indicated as shaded areas along the sequence of heavy chain (*left*) and light chain (*right*). Allotypic variations are indicated as vertical lines in the heavy chain. Clusters that were also detected in the media control samples are indicated as gray boxes. The detection of these peptides in the control samples can be explained by the use of human serum containing human IgG in the PBMC freezing media.

In the trastuzumab-treated samples, trastuzumab-derived peptides were detected multiple times and in several length variants clustering in sequence regions, increasing confidence in correctness of peptide identification. In addition, donors sharing the same HLA-DR alleles showed common peptide clusters. Lack of identification of trastuzumab-derived peptides in the negative control samples ruled out false positive identification of trastuzumab-derived peptides in the respective samples derived from the same donor. Clusters unique to KLH were detected in numerous regions of this large protein (Fig. S2). Clusters unique to trastuzumab were mainly identified from 2 regions of the trastuzumab sequence spanning residues 71 to 92 of the VH domain and residues 39 to 60 of the VL domain (Fig. 5). Moreover, clusters were identified with low frequency spanning 4 additional regions of sequence in the heavy and light chain constant domains, some of which were also detected in the media control samples at very low levels, which can be explained by the use of human serum containing human IgG in the PBMC freezing media; however, no peptides were found that spanned the allotypic variations and no changes in overall peptide pattern were observed in CH1 and CH3 in any of the donors. This is in line with data on 5 different marketed mAbs covering the G1m17, G1m1,17, G1m1,3 and G1m3 allotypes, for which no clusters were detected in the sequence regions with allotypic variations (unpublished data), suggesting that these regions are not presented in general, at least not to a detectable extent.

## Discussion

G1m allotyping was done by PCR amplification and sequencing, and the high-throughput nature of the screen facilitated the rapid allotyping of 570 samples at both CH1 and CH3 loci. The frequencies of G1m1,17 and G1m3 found in the panel of donors tested were in close agreement with the frequencies reported by serological methods.^22^ Within the panel tested we were also able to identify a number of genotypes that are rare in the world population: ^22^ individuals that were homozygous G1m17/G1m17 at their CH1 locus and heterozygous G1m1/nG1m1 at their CH3 locus or individuals that were heterozygous G1m17/G1m3 at their CH1 locus and homozygous nG1m1/nG1m1 at their CH3 locus.

An allotypic mismatch between therapeutic antibody and recipient has the potential to cause immunogenic reactions and could have wide-ranging consequences for the market of therapeutic antibodies. The aim of this study was therefore to investigate the effect of the IgG1 constant region allotype on the potential immunogenicity of therapeutic antibodies using allotypic variants of trastuzumab as a model. We measured the immunogenicity of trastuzumab using *ex vivo* T-cell assays with donors that were homozygous for the G1m3 allotype (the most frequent allotype in the European population) and the G1m1,17 allotype (dominant in some Afro-Caribbean, aboriginal, and Asian populations).^22^ The IgG1 allotypes differ by one amino acid in the CH1 region (G1m3 and G1m17) and by 2 amino acids in CH3 (G1m1 and nG1m1). *In silico* HLA class II binding analysis of the amino acid sequences in these regions by iTope™ ^31^ showed no significant differences in the HLA class II binding profiles in both the CH1 and CH3 regions (Fig. S3), suggesting that the allotypic differences would not affect T-cell responses.

In *ex vivo* T-cell assays, the proliferation and IL-2 ELISpot assay data show that a small proportion of donors were induced to produce positive T-cell responses against both antibody samples tested, regardless of whether or not the allotypes of the antibodies and donors were matched. The frequency of positive T-cell responses (SI ≥ 1.90, p < 0.05 in both proliferation and IL-2 ELISpot assays) of the matched homozygous G1m3 allotype donors to G1m3-trastuzumab was 5% in a study cohort of 37 donors, and for the matched homozygous G1m1,17 allotype donors a response rate of 3% to G1m1,17-trastuzumab was observed in a study cohort of 31 donors. In the cohort of 37 G1m3 allotype donors, stimulation with the mismatched G1m1,17-trastuzumab gave a combined assay response rate of 5%, and the inverse mismatch with G1m3-trastuzumab and homozygous G1m1,17 allotype donors (total cohort 31 donors) gave a positive response rate of 6% (Table 2). This small difference in responding donors between the matched/mismatched donor sets was not statistically significant (P = 0.735). This therefore suggests that stimulation of G1m3 or G1m1,17 trastuzumab with mismatched G1m3 or G1m1,17 allotype donor populations had no overall effect on the immunogenic potential of trastuzumab. Analysis of the sequence comprising the IgG1 allotypes in the CH1 and CH3 domains by using allotype mismatched and matched donors in T-cell epitope mapping studies revealed that peptides containing the allotypic substitutions did not stimulate an enhanced T-cell response in mismatched donors. Interestingly, an overlapping peptide (one amino acid C-terminal to the region containing the allotypic substitutions) that spans human germline sequence present in all allotypes in CH3 appears to contain a very weak cryptic T-cell epitope observed in a low number of donors.

The T-cell response rates observed with the allotypic variants of trastuzumab against allotype matched and mismatched donors correspond well with previous *ex vivo* time course T-cell assay studies using trastuzumab, which indicate that T-cell responses are reproducibly between 4 and 6% in study cohorts of mixed IgG1 allotypes.^25,29^ This correlates with clinical studies investigating the frequency of anti-infliximab and anti-adalimumab antibodies in patients with rheumatoid arthritis, which have shown no statistical correlation between the immunogenicity of either infliximab or adalimumab (both G1m1,17 allotype) and mismatched allotype patients.^23,24^ These and our results contrast with another study that investigated CD4+ T-cell responses to overlapping peptides spanning the G1m1 allelic variation, which identified a downstream T-cell epitope that was activated by asparaginyl endopeptidase cleavage at the G1m1 allelic variation and recognized by nG1m1 donors, especially those carrying HLA-DRB1*07; ^32^ however, studies with whole proteins, which would allow the full processing and presentation pathway to be taken into account, were not done. Further evidence for the lack of responses to IgG1 allotypes in mismatched donors was provided by the MAPPs assay, where neither peptides spanning the allotypic sequence variations nor generation of neo-agretopes downstream of the allotypic modification were found using DCs pulsed with both trastuzumab allotypes. This result also confirms that the weak T-cell epitope identified in our peptide T-cell assays is likely to be cryptic.

The frequencies of responding donors in the *ex vivo* T-cell assay and their reproducibility across studies suggests the presence of a weak T-cell epitope in trastuzumab and the pattern of responding donors suggests that this T-cell epitope is common to both IgG1 antibody allotypes, since a low number of donors (e.g., donors 24 and 25) responded in both proliferation and IL-2 ELISpot assays to the G1m3 and G1m1,17 antibody allotypes. It is clear that the common pattern of donors responding irrespective of matched or mis-matched G1m3 or G1m1,17 allotypes indicates the presence of a common T-cell epitope(s) that must be located outside of the region covering the IgG1 allotype sequences. Moreover, the frequency and magnitude of T-cell responses observed in both matched and mis-matched donors supports the fact that the common T-cell epitope(s) is likely to be very weak. Further evidence for this is provided by the MAPPs analysis where a major frequency cluster of peptides presented on the surface of DCs was identified in the VH domain regardless of G1m allotype, although the lower frequency cluster in the VL domain also has the potential to trigger low frequency T-cell responses.

In summary, these findings show that sequence differences between G1m3 and G1m1,17 allotypes do not appear to produce novel T-cell epitopes in mismatched individuals and that allotypic differences between IgG1 antibody therapeutics are unlikely to be a major factor in the generation of immune responses in the clinic.

## Material and methods

### Isolation of peripheral blood mononuclear cells

Healthy community donor buffy coats (from blood drawn within 24 hours) were obtained from National Blood Transfusion Service (Addenbrooke’s Hospital, Cambridge, UK) according to approval granted by Addenbrooke’s Hospital Local Research Ethics Committee. Peripheral blood mononuclear cells (PBMC) were isolated from buffy coats by Ficoll (GE Healthcare, Little Chalfont, UK) density centrifugation and CD8^+^ T cells were depleted using CD8^+^ RosetteSep^TM ^(StemCell Technologies, Inc., Grenoble, France). Donors were characterized by identifying HLA-DR haplotypes using an Allset^TM^ SSP-PCR based tissue-typing kit (Biotest, Solihull, UK) and a subset of donors selected for the T-cell assay study were further characterized, using a similar methodology, for HLA-DQ haplotype. T-cell responses to a control antigen, KLH (Pierce, Cramlington, UK) were assessed using fresh cells before aliquots of donor PBMC were frozen.

### Identification of donor G1m allotype

PBMC genomic DNA was purified using a DNeasy Blood and Tissue kit (Qiagen, Crawley, UK) as described by the manufacturer. IgG1 CH1 and CH3 domains were PCR amplified using domain-specific primers in conjunction with IgG1 hinge-specific primers (CH1for: 5′-ACCTCTGGGGGCACAGCG-3′ + CH1rev: 5′-TGAGTTTTGTCACAAGATTTGGG-3′ and CH3for: 5′-GAGCCCAAATCTTGTGACAA-3′ + CH3rev: 5′-GGCGATGTCGCTGGGA-3′) (Fig. S4). The amplified CH1 sequence encompassed the allelic variation at nucleotide position 359 and the amplified CH3 sequence encompassed the allelic variations at positions 36 and 40 (nucleotide numbering according to IMGT; www.imgt.org). All PCRs were performed according to the following regime: denaturing at 95 °C for 25 s, annealing at 57 °C for 25 s and extension at 72 °C for 50 s using Bio-X-Act™ polymerase (Bioline, London, UK), for 40 cycles.

PCR amplification products were purified prior to sequencing using Agencourt AMPure beads (Beckman Coulter Genomics, Takely, UK). Sequencing was performed using an ABI 3730XL DNA analyzer (Applied Biosystems Inc., Warrington, UK). CH1-specific 18-mer sequencing primer: 5′-CCTGGACTGGGGCTGCAT-3′ (CH1seq). CH3-specific 18-mer sequencing primer: 5′-GCCAAAGGTGGGACCCGT-3′ (CH3seq). Sequence analysis was performed using Lasergene SeqMan Pro software (DNAstar, Madison, WI).

### Subjects

For the Time Course T-cell assay, a cohort of 68 donors (31 donors with homozygous G1m1,17 alleles or 37 homozygous G1m3 IgG1 alleles) was identified. The homozygous donors for both allotypes were selected to best represent the number and frequency of the major HLA-DR allotypes expressed in the European population (http://www.ncbi.nlm.nih.gov/gv/mhc/main.fcgi?cmd=init). (Fig. 1).

For the peptide assay, a subset of the above cohort (22 homozygous G1m1,17 and 32 homozygous G1m3 donors) was selected together with 16 randomly selected heterozygous donors. For the MAPPs assay, due to limited cell numbers, a fresh cohort of 7 homozygous G1m1,17, 12 homozygous G1m3 donors) and 16 heterozygous donors was selected.

### Preparation of G1m3-trastuzumab and G1m1,17-trastuzumab

The variable chain genes for trastuzumab were synthesized (GeneArt AG, Regensburg, Germany) based upon the published sequence described by Carter et al.^33^ The VH and VL genes were subcloned into vectors expressing whole antibody heavy and light chains, respectively. The VH domain was cloned into vectors containing human heavy chain constant domains encoding either the G1m3 or G1m1,17 and regulatory elements to express whole IgG heavy chain in mammalian cells. Similarly, the VL domain was cloned into a vector for the expression of the human light chain (kappa) constant domains and regulatory elements to express whole IgG light chain in mammalian cells. Vectors for the expression of heavy chains and light chains were as originally described.^34^ MedImmune vectors have been further engineered simply by introducing an Epstein-Barr virus OriP element. To obtain IgG1 proteins, the heavy and light chain IgG expressing vectors were transfected into HEK293/EBNA mammalian cells using standard methods. IgG1 proteins were expressed and secreted into the medium. Harvests were pooled and filtered prior to purification. The IgG1 was purified using Protein A chromatography and stored in phosphate-buffered saline (PBS) pH 7.4 at concentration of 10 mg/ml.

### Ex vivo time course T-cell proliferation assay

Time course T-cell assays were performed as described previously.^35^ Briefly, CD8^+^ T cell-depleted PBMC from each donor were thawed, counted and viability assessed. Cells were revived in room temperature AIM V® culture medium (Invitrogen, Paisley, UK) before adjusting the cell density to 4 × 10^6^ PBMC/ml (proliferation cell stock). The concentration of each trastuzumab sample was adjusted by dilution in AIM V®culture medium to make a 100 μg/ml culture stock. KLH was stored as a stock solution at 10 mg/ml in water at −20 °C, and a 200 μg/ml KLH culture stock was made by diluting in AIM V® culture medium.

For each donor, bulk cultures were established in which 1 ml of proliferation cell stock was added to each well of a 24-well plate. 1 ml each trastuzumab culture stock was added to sample wells in the 24-well plates giving a final well concentration of 50 μg/ml. The remaining wells were used as untreated background or as a positive control to which, respectively, 1 ml of AIM V® culture medium or KLH culture stock was added (final concentration 100 μg/ml). Cultures were incubated for a total of 8 days in a humidified CO_2_ incubator with 5% CO_2_. On days 5, 6, 7 and 8, the cells in each well were gently resuspended and 3x 100 μl aliquots transferred to wells in a round bottom 96-well plate. The cultures were pulsed with 0.5 μCi ^3^[H]-thymidine (Perkin Elmer®, Beaconsfield, UK) in 100 µl AIM V® culture medium and incubated for a further 18 hours before harvesting onto filter mats (Perkin Elmer®, Beaconsfield, UK) using a TomTec Mach III cell harvester. Counts per minute (cpm) for each well were determined by Meltilex^TM^ (Perkin Elmer®, Beaconsfield, UK) scintillation counting on a Microplate Beta Counter in paralux, low background counting.

### IL-2 ELISpot assay

The donor cohorts used in the time course T-cell proliferation assay were also used to measure IL-2 secretion in ELISpot assays. ELISpot plates (Millipore, Watford, UK) were pre-wetted and coated overnight with 100 μl/well IL-2 capture antibody (R&D Systems, Abingdon, UK) in PBS. Plates were then washed twice in PBS, incubated overnight in block buffer (1% human serum albumin in PBS) and washed in AIM V® medium before use. Cells were thawed and revived as described above and the cell density adjusted to 5 × 10^6^ CD8^+^ T cell-depleted PBMC/ml in AIM V® culture medium.

Trastuzumab samples were diluted in AIM V® to a concentration of 100 μg/ml.

Trastuzumab samples (100 μl), were added to 100 μl cells and tested in sextuplicate cultures with negative control (AIM V® medium alone), no cells control and a phytohemagglutinin positive control (PHA, Sigma, Poole, UK) on each plate. After an 8 day incubation period, ELISpot plates were developed by sequential washing in dH_2_O and PBS (x3) prior to the addition of 100 μl filtered biotinylated detection antibody (R&D Systems, Abingdon, UK) in PBS/1% BSA. Following incubation at 37 °C for 1.5 h, plates were further washed in PBS (x3) and 100 μl filtered streptavidin-AP (R&D Systems, Abingdon, UK) in PBS/1% BSA was added for 1.5 hr at room temperature. The plates were then washed in PBS (4x), 100 μl BCIP/NBT (R&D Systems, Abingdon, UK) added to each well and incubated for 30 minutes at room temperature. Spot development was stopped by washing the wells and the backs of the wells 3 times with dH_2_O. Dried plates were scanned on an ELISpot Series 3b Analyser^TM^ (CTL-Europe Ltd, Aalen, Germany) and spots per well (spw) were determined using Immunospot^TM^ Version 4 software (CTL-Europe Ltd, Aalen, Germany).

### Epitope mapping

Seven 15mer peptides from human IgG1 spanning the allotypic variations of G1m3, G1m17, G1m1, nG1m1 and the peptide found by Stickler *et al* ^32^ to raise a T-cell response were synthesized (Abgent Europe Ltd, Oxford,UK) to >80% purity. The lyophilized peptides were resuspended in DMSO at a concentration of 10 mM. The peptide sequences were as follows:

1. **PSRD**E**L**TKNQVSLTC G1m1
2. **D**E**L**TKNQVSLTCLVK G1m1
3. TKNQVSLTCLVKGFY (ref ^32^)
4. PSR**E**E**M**TKNQVSLTC nG1m1
5. **E**E**M**TKNQVSLTCLVK nG1m1
6. VDK**R**VEPKSCDKTHT G1m3
7. VDK**K**VEPKSCDKTHT G1m17

PBMC were prepared as described above (section 2.8) and cell density was adjusted to 2.5 × 10^6^ cells/ml in AIM V® culture medium. Each peptide was tested at a final concentration of 5 μM in sextuplicate cultures for each donor. On day 7 post incubation with peptides, the cells were pulsed, harvested and counted as described in section 2.8.

### Data analysis

For both proliferation and IL-2 Elispot data sets, positive T-cell stimulation was defined by donors that produced a significant (p < 0.05) response above untreated controls (proliferation or IL-2 secretion), using a 2 tailed, one way Student’s T-test, with a stimulation index (mean test cpm or spw/mean untreated control cpm or spw) greater than 1.90 (SI ≥ 1.90). Positive T-cell responses are herein labeled as SI ≥ 1.90, p < 0.05. In addition, the data were analyzed to determine the coefficient of variance (CV) and standard deviation (SD). For the T-cell epitope mapping a background threshold was calculated from the mean number of positive (SI ≥ 1.90, p < 0.05) proliferation responses in the donor cohort plus twice the SD. Peptides that stimulated a high frequency of T-cell proliferation above the background response threshold were deemed to contain T-cell epitopes.

### MAPPs analysis

#### Generation of HLA-DR–specific beads for immunoprecipitation

MAb L243 specific for HLA-DRα was used to isolate HLA-DR–peptide complexes. Anti–HLA-DR antibody was immobilized on N-hydroxysuccinimide–activated beads (GE Healthcare Bio-Sciences AB, Uppsala, Sweden) according to the manufacturer’s protocol and stored containing 0.02% sodium azide. For confirmation of HLA-DR depletion efficiency of the L243-conjugated beads, cell lysates before and after immunoprecipitation with the beads were analyzed by Western Blotting using the non-overlapping HLA-DR–specific mAb 1B5 (Lifespan Biosciences, Seattle, Washington, USA).

#### Isolation of monocytes and preparation of immature dendritic cells

CD14+ cells were isolated from selected donor PBMC using human anti-CD14 microbeads (Miltenyi Biotech Ltd., Bisley, Surrey) by magnetic separation according to the manufacturer’s protocol. Monocytes were resuspended in warm AIM-V® medium containing 33 ng/mL GM-CSF (Peprotech, London, UK) and 3 ng/mL IL-4 (Peprotech, London, UK) to a final cell concentration of 0.3 × 10^5^ cells/mL and a cell number of 5 × 10^6^ cells per cell culture dish, and differentiated to immature dendritic cells for 5 days at 37 °C and 5% CO_2_.

#### Stimulation and loading of immature dendritic cells

On Day 5 of cell culture, immature DCs (typical yields >95% CD11c^+^, CD40^+^, CD80^+^, CD83^+^, CD86^+^ and HLA-DR^+^) were induced to maturation by adding lipopolysaccharide (Lipopolysaccharides from Salmonella enterica serotype abortus equi, Sigma, Poole, UK) to 1 µg/ml and loaded with the trastuzumab allotypic variants to a final concentration of 0.75 nmol/ml. After incubation for 24 hours at 37 °C and 5% CO_2_, dendritic cells were harvested and washed in PBS. After removal of liquid, the cell pellets were frozen at −70 °C.

#### Isolation of HLA-DR–associated peptides

For isolation of HLA-DR–associated peptides, dendritic cell pellets were lysed in hypotonic buffer containing 1% Triton X-100 and protease inhibitor tablets (Roche Diagnostics GmbH, Mannheim, Germany) for 1 hour at 4 °C on a horizontal shaker at 1100 RPM. After centrifugation, the lysate was incubated overnight at 4 °C with L243-conjugated beads for immunoprecipitation. After washing with wash buffer (PBS containing 0.5% Zwittergent) and several wash steps with distilled water, peptides were eluted from *HLA-DR* molecules by adding 0.1% trifluoracetic acid (Fluka, Buchs, Switzerland) at 37 °C and lyophilized using an Eppendorf Concentrator 5301 (Eppendorf AG, Hamburg, Germany).

#### Analysis of HLA-DR associated peptides

HLA-DR associated peptides were analyzed as previously described.^26-28^ Briefly, lyophilized peptides were resuspended in hydrophilic buffer containing 5% acetonitrile and 1.1% formic acid, and injected onto a self-packed fused-silica C18 reversed-phase nano-high-performance liquid chromatography column. Multiple injections were not performed due to limited amounts of sample. Peptide identification was performed using liquid chromatography (nano capillary system, Dionex Corporation, Sunnyvale, California, USA) on a reversed-phase column connected to a mass spectrometer (Q-Exactive, Thermo, California, USA) via electrospray ionization (LC-ESI-MS/MS). Chromatographic separation was achieved using a 5–80% acetonitrile gradient (buffer 1: 97.4% water, 2.5% acetonitrile, 0.1% formic acid; buffer 2: 17.4% water, 82.5% acetonitrile, 0.1% formic acid) and peptides were chromatographically separated using a 75 min gradient. Peptides were identified using a database search approach using the SEQUEST algorithm. Peptides with a delta mass of < 10 PPM to the expected mass, cross correlation values of XCorr > 2.3 for doubly charged ions, >2.8 for triply charged ions, >3.3 for quadruply charged ions, and a delta cross correlation >0.1, were considered as true hits.

## Supplementary Material

KMAB_A_1128605_s02.pdf
