# Supplementary material for: A comparison of the ability of the human IgG1 allotypes G1m3 and G1m1,17 to stimulate T-cell responses from allotype matched and mismatched donors
Source: MAbs. 2016 Jan 29;8(2):253–63. doi: 10.1080/19420862.2015.1128605 (PMC4966604; doi:10.1080/19420862.2015.1128605)
Supplement: KMAB_A_1128605_s02.pdf [file kmab-08-02-1128605-s001.pdf]

Supplementary Materials

Sequencing of CH1 and CH3 amplification products coupled to visual analysis of the returned chromatographs revealed the heterozygous allotype manifested itself as overlapping peaks corresponding to two different nucleotides each encoded by alternate G1m alleles (Supplementary Figure 1A). In contrast the homozygous allotype manifested itself as a single chromatograph peak, corresponding to two identical, overlapping G1m alleles (Supplementary Figure 1B).

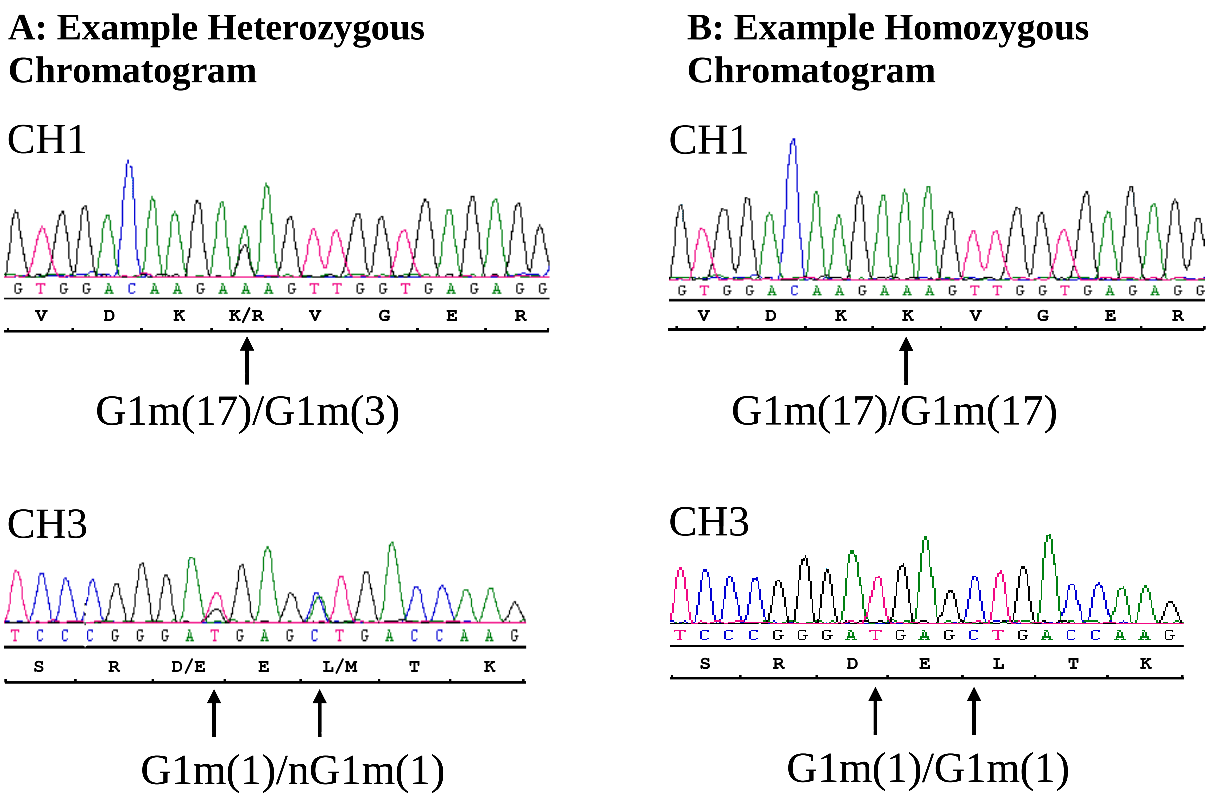

Supplementary Figure 1. Sequence Chromatographs Corresponding to (A) Heterozygote and (B) Homozygote Samples.

Overlapping peaks corresponding to alternately encoded alleles at either the CH1 or CH3 loci allow differentiation of heterozygote and homozygote donors. Polymorphic nucleotides specified by arrows. Single letter amino acid code is shown below the corresponding nucleotide triplet.

Supplementary Figure 2. HLA-DR–associated peptides produced by 5 x 10^6 dendritic cells from 35 different donors exposed to KLH. HLA-DR–associated peptides can originate from various regions of a protein and typically occur as multiple length variants which share the same HLA-DR binding core and form a “cluster”. Clusters are indicated as black boxes along the protein sequence.

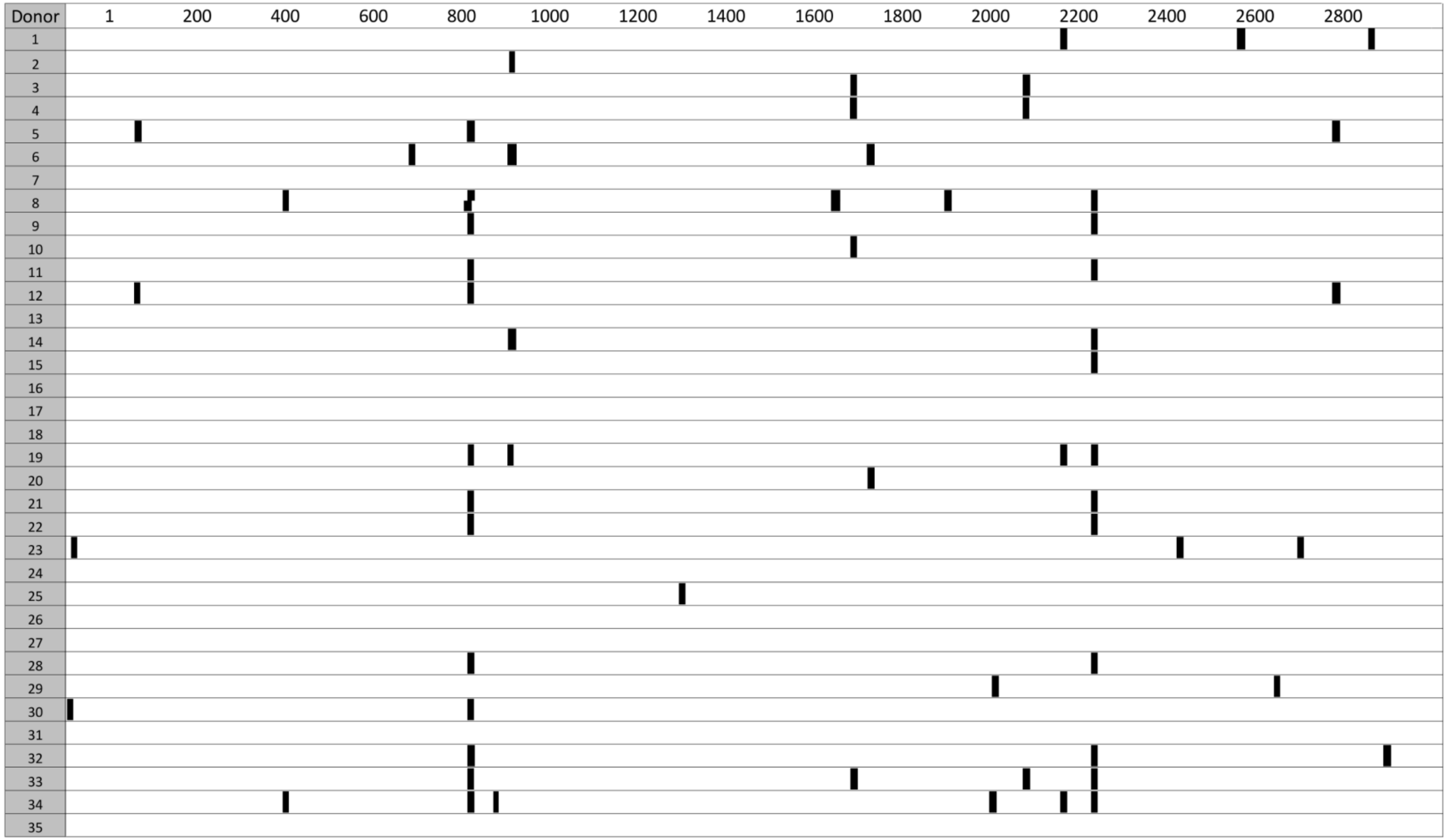

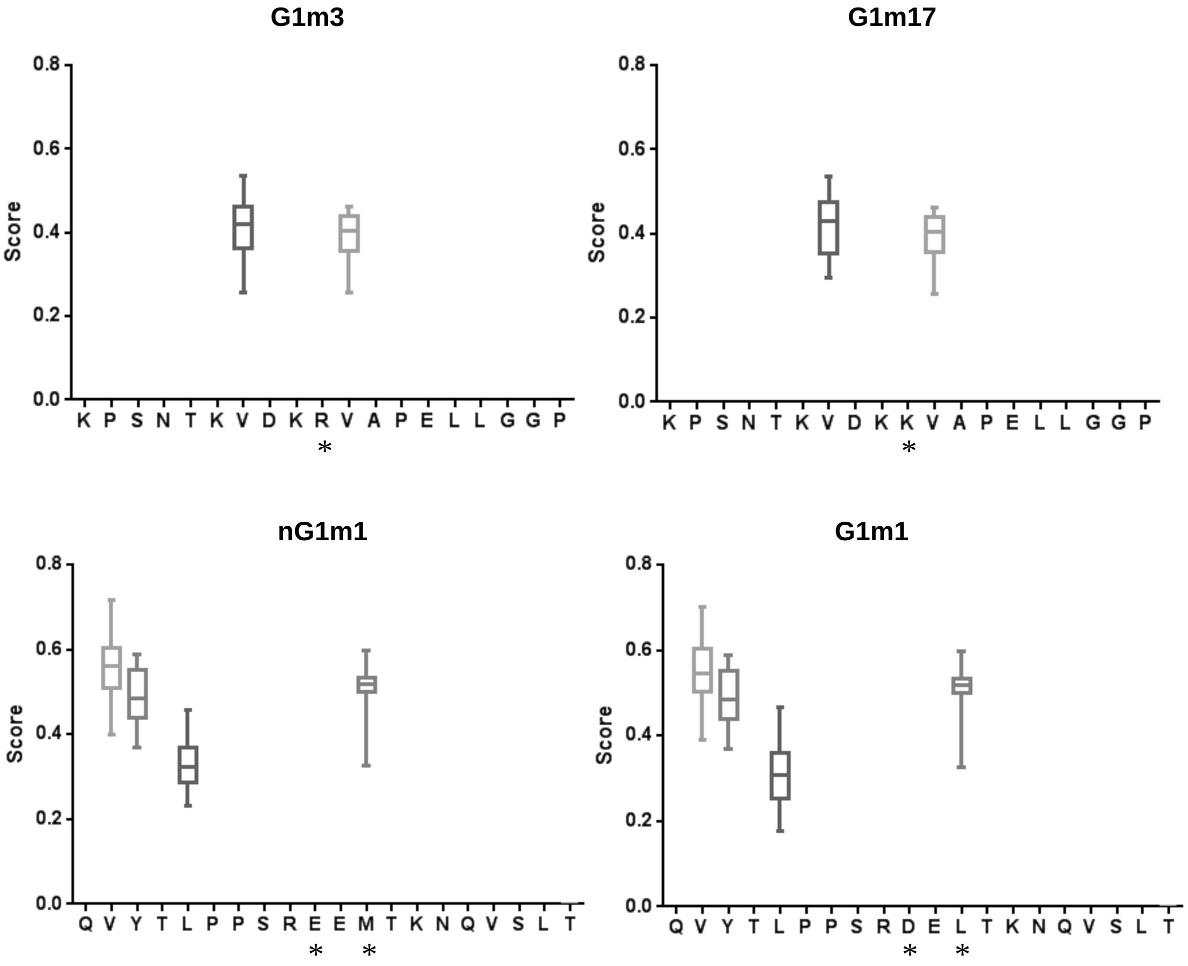

Supplementary Figure 3. *In silico* HLA-DR binding analysis of peptides spanning IgG1 allotypic variations (asterisked). Peptides spanning the selected sequence were tested as 9mer peptides in one amino acid increments against a panel of 34 HLA-DR alleles using iTope™ (Perry LC, Jones TD, Baker MP. New approaches to prediction of immune responses to therapeutic proteins during preclinical development. Drugs in R & D 2008; 9:385-96). The binding scores for each 9mer peptide are indicated above the the first amino acid of the peptide. The maximum and minimum scores are indicated, the boxed region represents the interquartile range and the median score is indicated by the line. Equivalent peptides that gave binding scores with each allotype were tested for significant differences using an unpaired, two-tailed Student's T test. No significant differences were observed ($p<0.05$).

Development of a G1m-specific CH1 and CH3 PCR-based Allotype Screen

A high throughput PCR-based allotype screen was developed that allowed discrimination of heterozygote and homozygote G1m(17)/G1m(3) and G1m(1)/nG1m(1) donor samples. Selective amplification of G1m CH1 and CH3 loci was achieved by designing PCR primers to regions of sequence diversity identified between the different human IgG isotypes (Supplementary Figure 3).

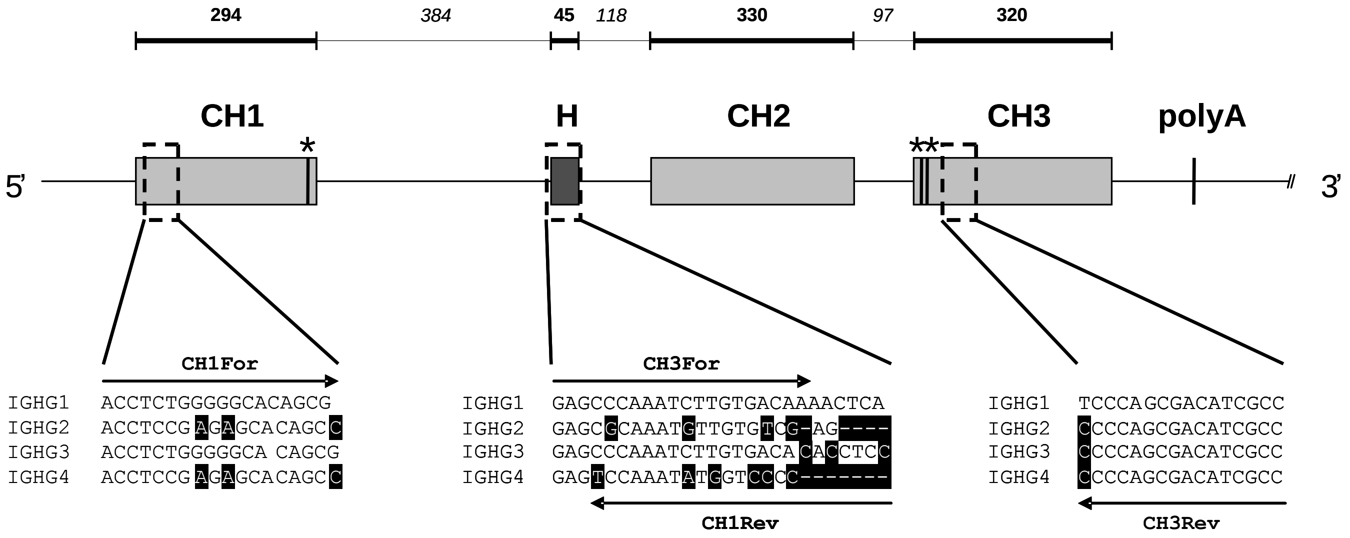

Supplementary Figure 4. G1m Constant Region Exon/Intron Organisation.

The length of exons (bold) and introns (italics) is given in base pairs. Dashed boxes define those regions of sequence to which the G1m specific primers anneal. Residues that differ from G1m nucleotide sequence at these positions are highlighted in black. Alignment generated using the ClustalW method. Primers are depicted as arrows with the tail and head of the arrow representing the 5' and 3' termini, respectively. H, hinge region. Asterisks (*) indicate position of allotype specific polymorphisms. Not drawn to scale. (Modified from IMGT Repetoire, http://www.imgt.org).

	Day 5				Day 6				Day 7				Day 8			
**Donor**	**Media**	**Trast. G1m3**	**Trast. G1m1,17**	**KLH**	**Media**	**Trast. G1m3**	**Trast. G1m1,17**	**KLH**	**Media**	**Trast. G1m3**	**Trast. G1m1,17**	**KLH**	**Media**	**Trast. G1m3**	**Trast. G1m1,17**	**KLH**
**2**	2909	2943	1619	4831	2209	2233	2088	4051	1669	1249	1478	1708	1669	1249	1478	1708
**3**	2840	2495	1666	3657	443	515	533	1892	1912	2019	2007	2426	1912	2019	2007	2426
**4**	1099	1511	1230	3350	269	346	402	3445	2118	2236	1494	2144	2118	2236	1494	2144
**5**	958	1093	1003	1629	848	908	914	3488	1478	1264	1028	3658	1636	1534	1821	6468
**7**	1211	1588	1769	8999	630	768	1044	11101	2970	3271	5166	20113	2963	4854	5372	14131
**9**	830	706	833	3274	790	722	774	7946	810	657	575	8304	593	410	664	10139
**10**	918	814	730	5036	610	614	806	6660	1196	807	744	7548	804	565	776	12929
**15**	3769	4026	4016	7730	2674	3253	1872	4596	2643	2573	1938	4925	2054	2306	1847	10731
**16**	2100	2630	1992	5222	1376	1567	552	1877	3602	5270	2713	10170	6629	7693	4991	15998
**17**	746	906	686	4035	1365	2116	1971	5182	1923	1600	1358	5460	1795	2057	1593	10008
**23**	842	932	1287	3576	540	641	989	2595	611	551	516	1498	1093	1366	1405	3955
**24**	867	1746	2025	6326	355	604	690	2743	934	994	1194	4225	1260	2165	2328	8350
**25**	383	703	649	2262	509	809	677	1761	469	456	385	3303	325	649	667	5502
**26**	2844	3378	2873	11810	3814	4601	4077	13221	3111	3363	2962	10595	2859	3279	3016	13131
**27**	3287	3402	3267	11271	2670	2920	1955	9689	5286	3612	4094	19576	6713	4515	4838	28535
**28**	750	1036	1035	1599	262	382	287	483	972	1339	898	2941	905	1401	1180	2955
**29**	693	1044	1227	7503	1435	1985	1879	13748	1119	1212	1106	14362	1169	1193	1818	22793
**33**	1520	1319	875	5012	2172	2088	1427	7394	2063	2461	3030	5615	1770	1842	1345	1454
**37**	1075	1466	1564	2188	1220	920	806	1431	740	1071	2429	1276	1172	1174	816	376
**39**	1161	1139	1057	3799	756	1148	1094	6263	1299	431	463	6319	1608	1588	1962	5519
**40**	1809	1518	1425	5176	1897	1396	1222	10915	855	803	643	5714	1641	2099	1887	4194
**41**	1752	1887	2344	2931	634	1041	972	3120	2591	2503	4059	3906	1683	2648	2168	3152
**42**	1376	1827	1689	3775	1328	1424	1234	7080	1258	1224	1290	2835	5410	4153	3018	1037
**43**	1509	2173	2028	4310	1090	1166	1030	5029	588	794	639	4768	1465	2407	2322	5674
**44**	1250	1744	1973	3685	1049	1574	1848	6676	1010	962	1306	6666	2189	2833	3443	4022
**45**	2578	4052	4441	4112	1388	1949	2044	5427	4464	2541	2708	3106	2239	2548	2490	4386
**48**	356	535	284	371	306	298	309	897	384	128	77	499	342	323	260	257
**49**	317	520	436	250	556	581	589	399	5420	4471	6165	6794	349	571	523	315
**52**	3229	3550	2934	4492	4121	2513	2051	8916	3665	1400	1578	5669	6184	2775	3985	2190
**54**	2290	3558	3667	5222	2043	1622	1562	7178	2602	1562	1303	3515	2137	2195	3468	2063
**55**	4779	3067	3318	6727	4362	2374	2004	5885	5280	1818	1142	2513	6764	4294	4122	3243
**56**	7646	8492	6515	7668	8393	6541	5329	9821	5093	3683	2689	3457	10941	6704	9108	2837
**57**	1865	2676	2385	4047	881	1084	1196	5635	4864	3296	7042	4443	939	868	983	6849
**58**	3735	5748	5688	5714	1782	1688	1667	5419	3265	3542	1623	3144	1193	1483	1935	6081
**59**	1983	2858	3249	4849	1475	1289	1313	8376	3188	2304	770	5151	2148	1407	1562	10156
**60**	3120	3883	4392	5406	796	1030	1376	6288	1765	391	486	3452	1401	1598	2000	12567
**61**	805	1243	1407	2291	873	1249	1279	4949	7390	1789	3618	4539	1541	1177	1141	4356
**66**	966	1234	1685	3573	801	1031	907	3077	323	342	500	1925	1459	1023	1274	5019

Supplementary Table 1. Average cpm values (to the nearest whole number) for G1m3 donors tested against both G1m3 and G1m1,17 trastuzumab (Trast.) for the time-course T cell assay (days 5 to 8).

	Day 5				Day 6				Day 7				Day 8			
Donor	Media	Trast. G1m3	Trast. G1m1,17	KLH	Media	Trast. G1m3	Trast. G1m1,17	KLH	Media	Trast. G1m3	Trast. G1m1,17	KLH	Media	Trast. G1m3	Trast. G1m1,17	KLH
1	1827	2591	2255	10352	1670	1914	2079	11504	1856	1498	1697	1374	1856	1498	1697	1374
6	1432	1540	1276	9149	1039	1339	1239	14570	2211	2333	1226	29792	2011	3043	1740	25014
8	3568	3661	3713	2693	522	590	753	433	6099	5388	5132	1188	6571	6473	6092	564
11	1708	1898	1529	6924	664	798	735	6213	2295	1781	1281	9819	2208	1827	1603	11686
12	1200	1384	1214	3189	134	193	228	826	1230	1139	996	2958	1385	1166	1037	5007
13	1947	2344	2134	6095	1277	1537	1630	6225	2025	2217	1497	7273	1971	1863	2149	7127
14	1139	957	1972	3773	1132	1466	2074	6900	3332	1724	5200	5849	2944	1365	4550	4480
18	530	408	616	1695	2537	2118	2819	7134	3019	1856	1940	4927	2476	2387	2301	8498
19	858	1476	1499	12593	876	1129	1416	10896	1398	1987	2325	24340	1305	1990	2980	27407
20	1562	2077	2700	7766	346	423	391	2671	1608	1518	1315	20386	1700	1494	1763	45674
21	1132	2068	1679	4196	1442	5071	1917	7009	1060	1467	1013	2275	1312	1433	2037	4189
22	2925	2996	1784	3970	3727	3937	2586	3496	2327	1271	676	875	5930	4341	2703	1273
28	750	1036	1035	1599	262	382	287	483	972	1339	898	2941	905	1401	1180	2955
30	675	699	582	3028	1031	956	753	1666	1088	712	640	1747	1062	629	803	2593
31	939	1655	1478	8775	1480	1535	1024	6159	1522	2617	1644	9851	1396	2303	1984	5220
32	312	466	424	1525	99	113	146	350	450	571	346	669	433	284	137	356
34	294	284	131	82	514	327	163	142	86	78	507	87	455	431	478	63
35	738	896	736	7555	1170	1473	926	14167	894	1252	653	9081	1354	2307	1501	3259
36	1282	1784	1217	4769	2035	3100	1633	7320	1616	2753	1328	5865	2651	4065	2129	2210
38	2557	1511	1375	5525	2478	1005	999	5807	2925	506	699	1331	3087	1863	2000	785
45	2578	4052	4441	4112	1388	1949	2044	5427	4464	2541	2708	3106	2239	2548	2490	4386
47	1160	1906	1368	2566	1084	1266	806	3098	1508	1091	547	2456	1467	2572	1475	3936
50	467	346	355	388	491	288	410	315	419	501	461	200	648	391	389	202
51	1964	3662	3355	4043	1652	2080	2096	6306	2071	1425	1158	5401	1823	3476	3279	3732
53	1755	1674	2447	1894	1492	771	905	1364	4484	2441	4064	8716	1493	1148	836	666
62	2530	2043	1882	4189	4339	2612	1953	7103	3014	2228	1640	2885	2271	3368	2335	1720
63	1349	1653	1839	2317	1505	1593	1517	3269	1255	851	730	2463	1734	1838	2231	5175
64	1427	1199	746	5990	1464	1645	1129	9515	958	1111	706	4606	1210	1572	1436	2149
65	1505	1676	1168	5999	1977	1940	1524	17909	1532	2125	8479	16059	1710	1828	1487	14384
67	1013	1065	1068	3626	799	1041	829	5054	663	565	508	4471	788	1579	1608	4733
68	1937	2221	2067	4407	2080	2021	1961	5649	1099	863	768	3997	2057	2423	2903	5552

Supplementary Table 2. Average cpm values (to the nearest whole number) for G1m1,17 donors tested against both G1m3 and G1m1,17 trastuzumab for the time-course T cell assay (days 5 to 8).

Donors: G1m3				
Donor	Media	Trast. G1m3	Trast. G1m1,17	KLH
2	4.33	4.50	4.83	57.50
3	2.50	3.00	3.00	18.25
4	4.50	4.67	4.67	25.00
5	0.17	2.17	5.67	10.25
7	15.50	20.00	20.83	50.50
9	6.17	5.50	6.17	43.25
10	5.83	6.33	7.00	20.75
15	33.67	37.67	31.50	12.75
16	18.50	13.17	2.33	39.25
17	9.00	6.17	5.83	31.25
23	43.67	51.33	36.50	41.25
24	9.67	19.17	21.33	89.75
25	9.17	20.40	24.83	32.50
26	16.40	13.33	11.50	36.50
27	41.33	37.67	52.00	119.50
28	8.83	11.67	9.33	33.75
29	4.00	1.83	1.83	24.75
33	9.83	11.67	14.17	86.00
37	16.67	13.17	16.83	40.25
39	4.00	1.83	3.50	21.50
40	27.00	17.17	19.83	123.50
41	87.17	92.75	144.75	140.75
42	231.67	263.50	260.50	443.50
43	20.80	19.20	3.83	121.50
44	57.00	65.50	82.83	151.75
45	59.50	86.00	88.83	115.00
48	0.33	0.00	0.17	0.75
49	ND	ND	ND	ND
52	210.33	265.00	297.33	461.25
54	174.60	322.83	295.17	505.25
55	70.50	31.67	20.50	78.25
56	181.33	136.50	143.50	278.50
57	483.67	437.33	598.83	1092.75
58	410.50	454.67	516.83	800.25
59	385.00	514.50	304.17	440.75
60	501.67	586.33	648.33	617.00
61	130.67	148.67	207.17	727.25
66	75.50	121.00	72.40	241.75

Donors: G1m1,17				
Donor	Media	Trast. G1m3	Trast. G1m1,17	KLH
1	2.67	3.67	4.00	14.00
6	1.00	9.50	10.50	43.00
8	8.17	12.33	11.00	39.50
11	18.00	16.67	27.33	49.25
12	17.50	21.17	17.67	22.50
13	41.17	18.50	21.83	102.25
14	53.50	58.33	59.00	180.75
18	2.83	1.50	3.33	17.50
19	3.17	4.67	7.50	13.25
20	2.00	1.17	1.17	25.00
21	8.17	12.33	10.50	20.75
22	7.00	12.17	11.83	45.25
28	8.83	11.67	9.33	33.75
30	4.00	1.33	4.83	25.25
31	33.00	25.67	15.83	68.25
32	ND	ND	ND	ND
34	0.33	0.17	0.83	0.25
35	21.83	39.67	15.17	42.67
36	17.40	19.33	15.33	33.33
38	7.50	7.83	11.33	37.00
45	59.50	86.00	88.83	115.00
47	82.83	55.17	52.50	56.00
50	0.33	0.17	0.17	2.00
51	209.67	487.50	301.50	450.50
53	91.67	90.67	61.50	94.75
62	130.80	98.17	141.33	444.75
63	265.17	374.83	392.00	603.25
64	272.40	205.33	271.50	575.25
65	94.33	77.17	118.50	199.00
67	152.50	354.83	260.25	107.75
68	430.50	503.00	460.83	379.00

Supplementary Table 3. Average spw values for homozygous donors (either G1m3 or G1m1,17) donors tested against both G1m3 and G1m1,17 trastuzumab (Trast.) for IL-2 ELISpot assay. ND = no data.
